# Plastic-scale-model assembly of ultrathin film MEMS piezoresistive strain sensor with conventional vacuum-suction chip mounter

**DOI:** 10.1038/s41598-019-39364-2

**Published:** 2019-02-13

**Authors:** Seiichi Takamatsu, Shintaro Goto, Michitaka Yamamoto, Takahiro Yamashita, Takeshi Kobayashi, Toshihiro Itoh

**Affiliations:** 1 The University of Tokyo, Graduate School of Frontier Sciences, Kashiwa, 277-8563 Japan; 2 National Institute of Advanced Industrial Science and Technology, Tsukuba, 305-8564 Japan; 3 NMEMS Technology Research Organization, Chiyoda, Tokyo, 101-0026 Japan

## Abstract

We developed a plastic-scale-model assembly of an ultrathin film piezoresistive microelectromechanical systems (MEMS) strain sensor with a conventional vacuum-suction chip mounter for the application to flexible and wearable strain sensors. A plastic-scale-model MEMS chip consists of 5-μm ultrathin piezoresistive strain sensor film, ultrathin disconnection parts, and a thick outer frame. The chip mounter applies pressure to the ultrathin piezoresistive strain sensor film and cuts the disconnection parts to separate the sensor film from the outer frame. The sensor film is then picked up and placed on the desired area of a flexible substrate. To cut off and pick up the sensor film in the same manner as with a plastic scale model, the design of the sensor film and disconnection parts of MEMS chips were optimized through numerical simulation and chip-mounting experiments. The success rate of the 5-μm ultrathin sensor film mounting increased by decreasing the number and width of the disconnection parts. For a 5-μm-thick 1 × 5 mm^2^ sensor film, 4 disconnection parts of 20 μm in width achieved 100% success rate. The fabricated ultrathin MEMS piezoresistive strain sensor exhibited a gauge factor of 100 and high flexibility to withstand 0.37 [1/mm] bending curvature. Our plastic-scale-model assembly with a conventional vacuum-suction chip mounter will contribute to more practical manufacturing of ultrathin MEMS sensors.

## Introduction

Ultrathin microelectromechanical systems (MEMS) sensors, which are made of very thin (<5 μm thick) functional silicon film have been applied to healthcare monitoring^1,2^, structural health monitoring of infrastructure, and automotive, aircrafts, and other transportation equipment^3–5^. The advantages of ultrathin (<5 μm thick) MEMS devices are highly flexibility, long-term stability, high sensor sensitivity, and mass productivity because ultrathin film exhibits smaller bending strain than conventional thick MEMS films^1,6,7^. Previous studies on ultrathin MEMS sensors reported ultrathin piezoelectric vibration sensors^3^, pressure sensors^1^, photodiodes^2^ and strain sensors^4,5^. Among these sensing devices, ultrathin MEMS strain sensors are promising sensing device for wearable and flexible physical sensor applications because they are not only highly sensitive but also flexible in comparing with conventional strain gauges. Existing strain sensors are metal strain gauges and semiconductor strain gauges and are used for all kinds of physical sensors including force, tension, weight, pressure, deformation, and vibration sensors. Metal strain gauges are flexible because they are made of thin copper-nickel alloy foil but exhibits small gauge factor of 2 while semiconductor strain gauge has large gauge factor of 100 but it is easily broken by bending because more than 300-μm thick brittle semiconductor silicon was used as a substrate^8^. Therefore, ultrathin silicon strain sensors will offer highly sensitive strain gauges with high flexibility for the application to wearable and flexible physical sensors.

**Figure 1:**
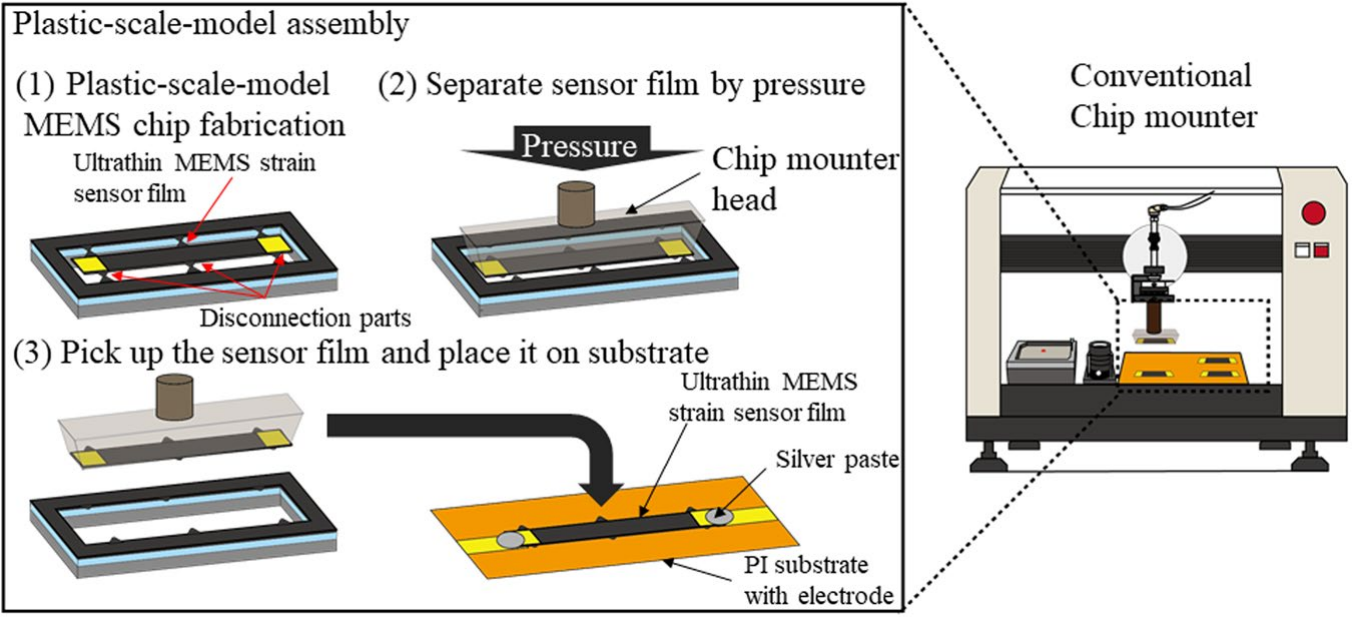
Schematic view of Plastic-scale-model assembly of ultrathin film MEMS piezoresistive strain sensor with conventional vacuum-suction chip mounter.

The assembly of ultrathin MEMS sensors, which involves releasing the ultrathin MEMS functional film and transferring the ultrathin film onto a flexible substrate, has been difficult. Previous studies^1,2,6,7,9–11^ on ultrathin-MEMS-sensor assembly reported on the surface micromachining MEMS structure as a ultrathin MEMS-film-releasing structure. The surface micromachining MEMS structure consists of ultrathin silicon film, etched silicon dioxide, and backside silicon substrate. In this structure, ultrathin silicon film weakly adheres to the backside silicon substrate with an etched silicon dioxide layer. The ultrathin-MEMS-film-transfer method involves a Polydimethylsiloxane (PDMS) stamp because PDMS is adhesive and soft^6,7,10^. After the PDMS stamp adheres to the ultrathin MEMS film and breaks the etched silicon dioxide layer to release the ultrathin MEMS film from the backside silicon substrate, the MEMS film is moved to the flexible substrate. However, the PDMS-based ultrathin-MEMS-film transfer is not compatible with conventional chip mounters since such mounters use a vacuum-suction chip-mounting head. To develop a ultrathin-MEMS-film-stain-sensor assembly for conventional semiconductor factories, we developed a plastic-scale-model assembly for ultrathin-MEM-film-strain sensors by using a conventional vacuum-suction chip mounter^3–5,12^. With our plastic-scale-model assembly, we fabricated a plastic-scale-model MEMS chip, which utilizes silicon-on-insulator (SOI) wafer and consists of ultrathin MEMS strain sensor film, disconnection parts, and an outer frame, and the ultrathin MEMS strain sensor film is cut from the outer frame by breaking the disconnection parts with the conventional vacuum-suction chip mounter. Figure 1 shows our plastic-scale-model assembly for ultrathin film MEMS strain sensors. Firstly, we fabricate a plastic-scale-model MEMS chip which consists of 5-μm ultrathin piezoresistive strain sensor film, ultrathin disconnection parts, and a thick outer frame. After the vacuum-suction chip-mounting head pushes on the ultrathin MEMS strain sensor film and breaks the disconnection parts, the MEMS strain sensor film is picked up by vacuum suction and moved to the desired spot on a flexible plastic substrate. Our plastic-scale-model assembly with the conventional vacuum-suction chip mounter has advantages of low introduction cost of fabrication tools and stable adhesive force to pick up an ultrathin MEMS strain sensor film because the PDMS-stamp tools are not conventional and PDMS gradually loses its adhesive force. Because the conventional vacuum-suction chip mounter picks up mechanically strong MEMS bare chips of more than 300 μm thick, handling mechanically weak ultrathin MEMS strain sensor film is difficult, breaking the sensor film. Our plastic-scale-model assembly also requires not only picking up the ultrathin MEMS strain sensor film but also separating the disconnection parts at the same time. When pressure to separate the disconnection parts is applied to the sensor film, high pressure is applied to the ultrathin MEMS strain sensor film, resulting in breaking of the sensor film. We thus previously reported^3–5^ the low success rate of chip-mounting.

To solve the problem of ultrathin-sensor separation, we made a mechanical model of ultrathin-MEMS-sensor-film separation and optimized the design of ultrathin MEMS strain sensor film and disconnection parts to achieve high yield of ultrathin MEMS sensor film mounting with a conventional vacuum-suction chip mounter. Specifically, we analyzed the pressure and strain distribution on an ultrathin MEMS sensor film and disconnection parts under separation pressure. We made a simple theoretical model of a plastic-scale-model MEMS chip to find key parameters of disconnection-part geometry. We then conducted a finite element model (FEM) simulation of the plastic-scale-model MEMS chip with different geometry design in which key parameters of the disconnection part were changed. Then, we conducted ultrathin MEMS strain sensor film mounting experiments with a conventional vacuum-suction chip mounter after we fabricated the plastic-scale-model MEMS chip with the same geometry. The optimal design of the plastic-scale-model MEMS chip for plastic-scale-model assembly was finally determined. To measure strain, the chip had a piezoresistor which was p-ion-implanted n-type silicon film and changed its electric resistance according to applied strain. We also evaluated the piezoresistive stain sensitivity of the fabricated ultrathin MEMS strain sensor.

## Results

### Structure of plastic-scale-model MEMS chip and fabrication process

The structure of the plastic-scale-model MEMS chip is shown in Fig. 1. The ultrathin MEMS strain sensor film is only 5 μm thick while conventional piezoresistive MEMS strain sensors are more than 100 μm thick. The strain sensing material is a piezoresistor formed on the surface of 5 μm thick device silicon layer. Phosphor-ions are implanted on n-doped silicon layer to form 100 nm thick piezoresistor. The piezoresistive effect of silicon exhibits higher electric resistance change under applied strain than conventional metals^8^. The plastic-scale-model MEMS chip is made of an SOI wafer, and the thickness of the ultrathin MEMS strain sensor film is determined by the 5-μm-thick device silicon layer of the SOI wafer. The substrate is a conventional flexible printed circuit board consisting of polyimide (PI) film and a copper (Cu) electrode. The ultrathin MEMS strain sensor film is attached to the PI film with glue and the sensor film and Cu electrode on the PI film are connected using stretchable silver paste. Our plastic-scale-model assembly consists of the following three parts:

1. A MEMS fabrication process to fabricate plastic-scale-model MEMS chip containing ultrathin MEMS strain sensor film, disconnection parts, and an outer frame, which is a similar structure to a plastic-scale-model before assembly.
2. separation of ultrathin MEMS strain sensor film from the outer frame and mounting the sensor on the substrate with a conventional vacuum-suction chip mounter, which is similar to the separation of the plastic model parts from the outer frame.
3. picking up of ultrathin MEMS sensor film by vacuum suction and placing the sensor film onto the flexible substrate.

### Analytic model of ultrathin MEMS strain sensor film mounting with conventional vacuum-suction chip mounter and key parameters

In previous studies^3–5^, the ultrathin MEMS sensor film breaks when the sensor film is separated from the outer frame since the pressure is applied not only to the disconnection parts of the MEMS chip but also the sensor film itself. To avoid breaking the sensor film, the pressure and strain distribution on the sensor film and disconnection parts under separation pressure were analyzed and a suitable design of a plastic-scale-model MEMS chip for sensor film-mounting were investigated.

**Figure 2:**
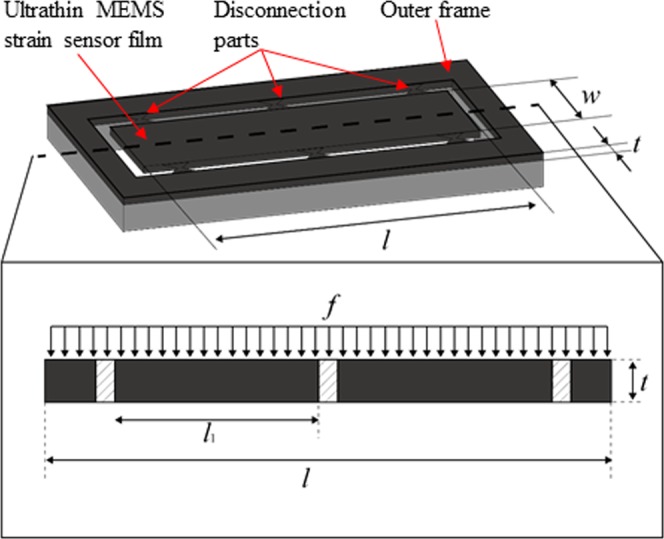
Simplified structure model of MEMS sensor chip.

Figure 2 shows a simplified structure model of a plastic-scale-model MEMS chip. The cross-sectional view of the MEMS chip is an ultrathin MEMS strain sensor film with several fixing supports, which are disconnection parts. Uniformly distributed load is applied to the sensor film by the chip mounter head and shear stress is concentrated on the disconnection parts. The load applied from the chip-mounter head induces a moment around the disconnection parts, which breaks the sensor film. The shear stress on the disconnection parts and the bending stress on the sensor film around the disconnection parts are modeled by changing the number and width of the disconnection parts and thickness, width, and length of the sensor film. The parameters of the disconnection parts and sensor film are as follows.

Load on the sensor film: *f*

Sensor film thickness: *t*

Sensor film length: *l*

Sensor film width: *w*

Distance among disconnection parts: ${\ell }_{1}$

Number of disconnection parts: *n*

Width of disconnection parts: *d*

When the uniformly distributed load is applied to the sensor film,

the shear stress per disconnection part *τ* is

1 $$\tau =\frac{fw\ell }{2ndt}$$

Considering the sensor film between disconnection parts, the bending moment around the disconnection parts *M* is.

2 $${\rm{M}}=\frac{f{{\ell }_{1}}^{2}}{12}.$$

The stress on the sensor film at the nearest point of the disconnection parts *ρ i*s

3 $${\rho }=\frac{Mt}{2{\rm{I}}}$$

Here, *I* is the cross-sectional second moment of the sensor film

4 $${I}=\frac{w{t}^{3}}{12}$$

According to Equations (3) and (4),

5 $$\rho =\frac{6M}{w{t}^{2}}$$

According to Equations (2) and (5),

6 $$\rho =\frac{f{{\ell }_{1}}^{2}}{2w{t}^{2}}=\frac{nd{l}_{1}^{2}}{{w}^{2}tl}\tau $$

The shear stress when the sensor film is separated from outer frame silicon is *τ* = *τ*_B_, where *τ*_*B*_ is the rupture stress of single crystal silicon, which is a constant value defined by the material property of silicon. Then, maximum stress on the sensor film *ρ* is defined as *ρ*_*M*_. In addition, the rupture stress of silicon is *ρ*_*B*_. If the sensor film is separated from the outer frame without breaking the senor film, the following inequation should be managed.

7 $${\rho }_{M} < {\rho }_{B}$$

In other words,

8 $$\frac{nd{l}_{1}^{2}}{{w}^{2}tl}{\tau }_{{\rm{B}}} < {\rho }_{{\rm{B}}}$$

9 $$\frac{nd{l}_{1}^{2}}{{w}^{2}tl} < \frac{{\rho }_{B}}{{\tau }_{B}}$$

Therefore, the condition of separating the sensor film without breaking the sensor film is that the left item of Inequation (9) should be small. To make this item small, *w*, *l*, *t*, *d*, ${\ell }_{1}$, and *n* are the key parameters of the plastic-scale-model MEMS chip geometry. Among these parameters, *w* and *l* cannot be changed because these geometries are defined by the strain sensor application. For separation of the sensor film, *d*, *n*, and ${\ell }_{1}\,$can be changed because the geometry of the disconnection parts does not affect the sensor film design. Therefore, we changed *d* and *n* and analyzed the mechanical model of ultrathin MEMS strain sensor film mounting.

### Comparison between numerical and experimental analysis of ultrathin MEMS strain sensor film mounting with conventional vacuum-suction chip mounter

We conducted FEM analysis of ultrathin MEMS strain sensor film mounting, especially of sensor film separation by changing the *d* and *n* of a plastic-scale-model MEMS chip with Ansys Workbench (Ansys Inc.). The ultrathin MEMS strain sensor film had an area of 1 × 5 mm^2^, which is the same as the device fabricated in the previous study^3–5^. Among the geometries of the MEMS chip, the number of disconnection parts: 2*n* varied from 4, 6, and 8. The width of the disconnection parts: *d* varied from 20 to 100 μm. The numerical simulation conditions were as follows:

1. Analytical model: Static structure analysis.
2. Analysis setting: The outer frame of the MEMS chip was fixed, and a uniformly distributed pressure of 12 kPa was loaded on the sensor film. The distributed pressure of 12 kPa was obtained from the experiment where the fabricated MEMS sensor chip was pressed by the chip mounter head.
3. Mesh: Automatic mesh and high goodness of fit (100).
4. Mechanical property of the structural material: Single crystal silicon (Young’s modulus and Poisson ratio were 185 GPa and 0.28, respectively).

**Figure 3:**
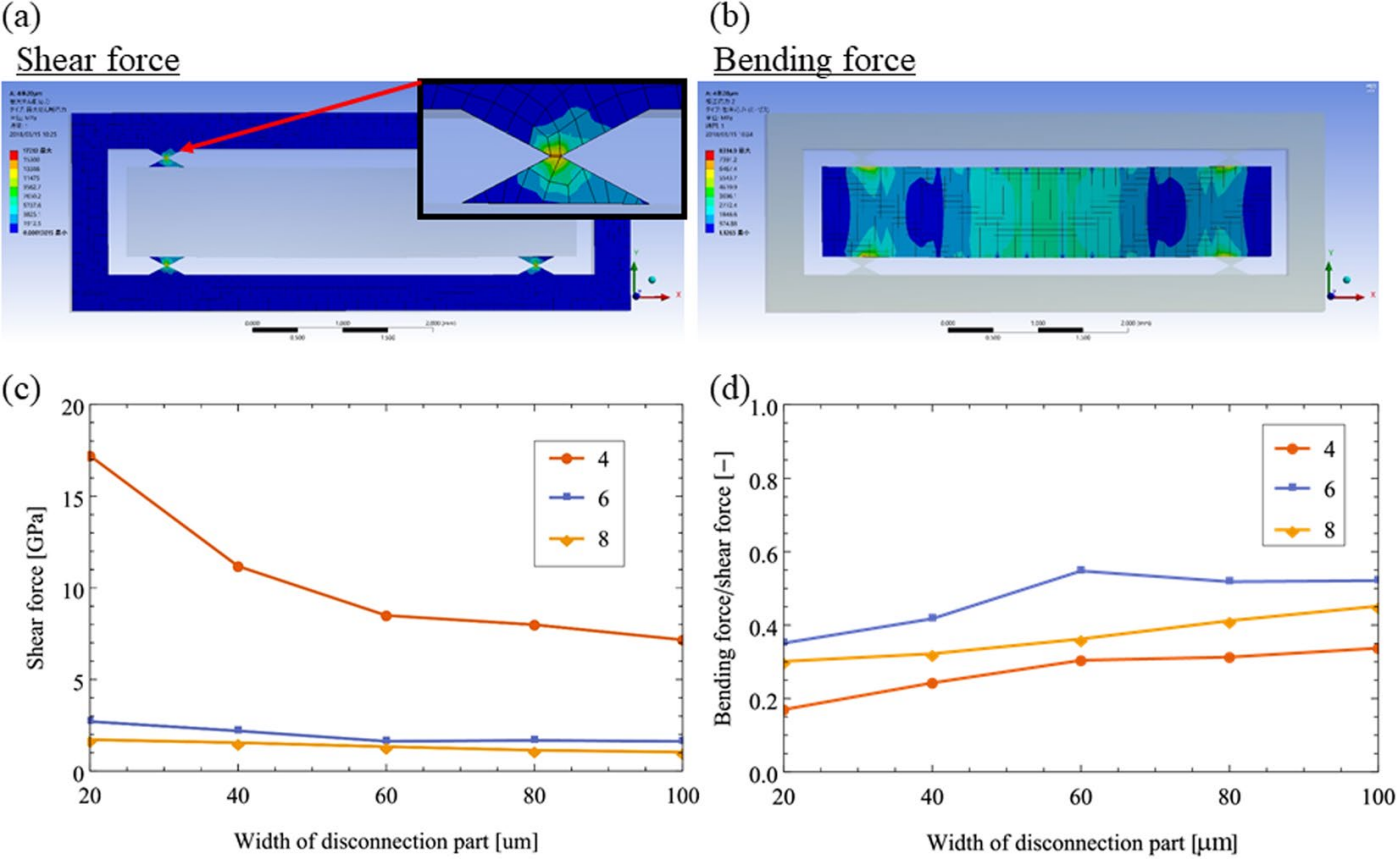
FEM results of chip-mounting of ultrathin MEMS strain sensor film.

Figure 3(a) shows the resultant stress concentration on the disconnection parts to cut the sensor film from the outer frame and the stress distribution on the sensor film. To cut the disconnection parts, the shear stress induced by the chip-mounter head should be concentrated only on the narrowest area of a disconnection part. Figure 3(a) shows that the shear stress concentrated in the narrowest point of a disconnection part to cut the sensor film from the outer frame. Figure 3(c) shows that the shear stress concentration increased when *n* decreased from 8 to 4. Specifically, when *d* was 20 μm, the concentrated shear stresses with 4, 6, and 8 disconnection parts were 1.7, 2.5, and 17 GPa, respectively. The decrease in *d* also increased the shear stress. For 4 disconnection parts, the shear stresses to the narrowest area of that part with *d* of 100, 80, 60, 40, and 20 μm were 7.1,7.9, 8.4, 11.2, and 17.2 GPa, which shows that the shear stress becomes concentrated by decreasing *d*. Therefore, a small *n* and narrow *d* are suitable to cut the ultrathin MEMS strain sensor film from the outer frame.

On the other hand, to avoid breaking the ultrathin MEMS strain sensor film, we also evaluated the bending moment on the sensor film, as shown in Fig. 3(b,d). Figure 3(b) shows the bending stress induced by the pressure of the chip mounter head concentrated on the area of the sensor film around the disconnection parts. Figure 3(d) shows the normalized bending stress, which is the ratio of the largest bending stress on the sensor film/shear stress on disconnection parts because the shear stress increases upto constant silicon rupture stress when the sensor film is cut off. It should be noted that the bending stress on the sensor film is required to be normalized because *f* in the numerical analysis was assumed a constant value of 12 kPa, but *f* for cutting the disconnection parts changes depending on the dimension of the MEMS chip geometry. Figure 3(d) shows that the bending stress/shear stress of 4 disconnection parts was smallest, which prevented the sensor film from breaking. For 4 disconnection parts, by narrowing *d* from 100 to 20 μm, the ratio decreased to 0.17. For 6 and 8 disconnection parts, the bending stress/shear stress also decreased by narrowing *d*. Thus, 4 disconnection parts with the small *d* (<40 μm) exhibits a small ratio between bending stress/shear stress and is suitable for sensor film separation without breaking the sensor film.

**Figure 4:**
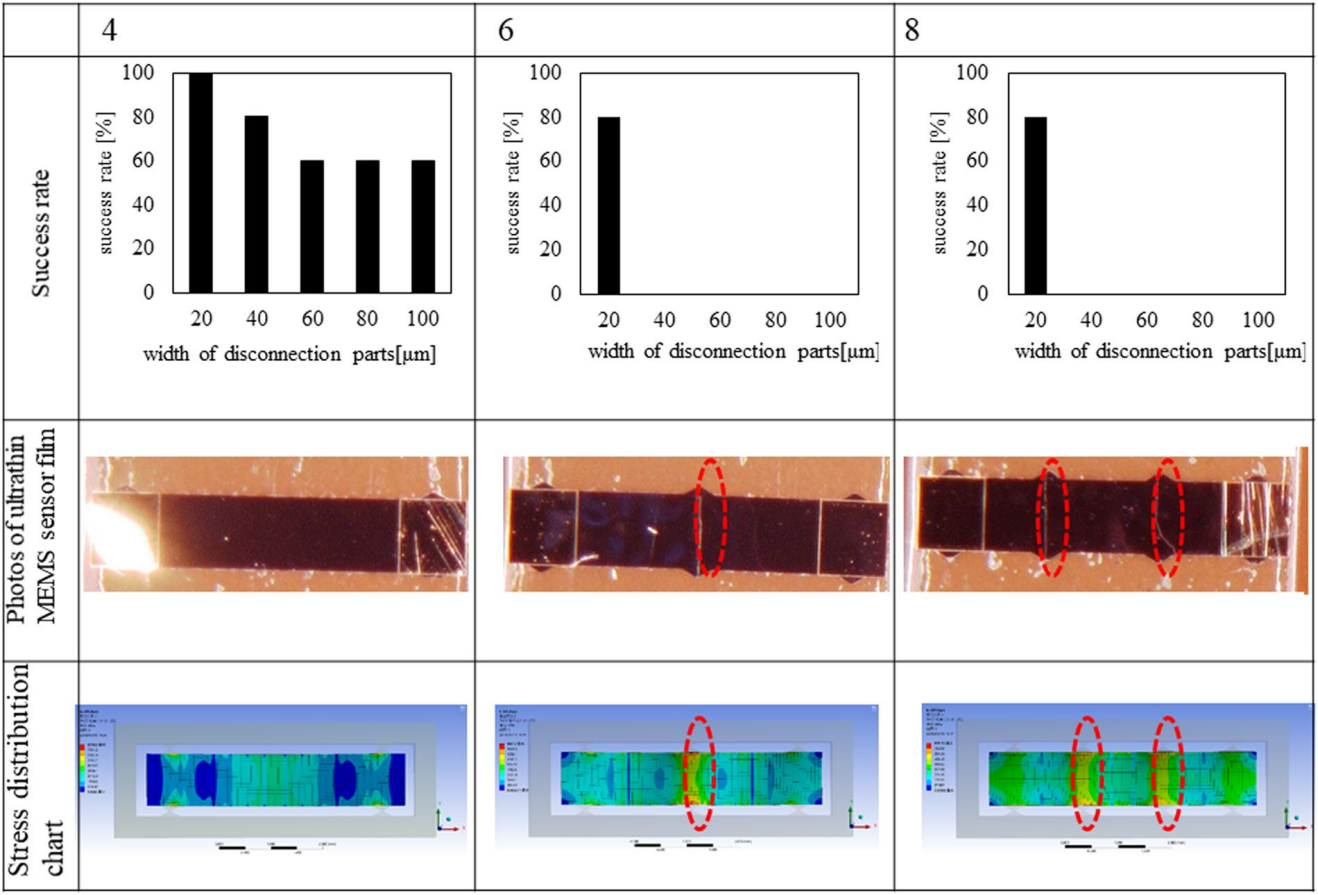
Experimental results of chip-mounting of ultrathin MEMS strain sensors.

We conducted the experimental analysis by fabricating the same geometrical design of plastic-scale-model MEMS chips as that used in the numerical analysis and separating and mounting the sensor film. Figure 4 shows the experimental results of the success rates of sensor film mounting and the resultant photographs of ultrathin MEMS sensor film mounted on the PI films. The MEMS chips with 4, 6, and 8 disconnection parts whose *d* ranged from 20 to 100 μm were used, five MEMS chips for each design were tested using a conventional vacuum-suction chip mounter (Hisol Model 400, Hisol corporation), and the success rate was evaluated. Figure 4 also shows the optimal design of MEMS chips and the disconnection parts. The 1 × 5 mm^2^ ultrathin MEMS strain sensor film had 4 disconnection parts with *d* of 20 μm because its success rate was 100%. By narrowing the *d* of the 4 disconnection parts from 100 to 20 μm, the success rate increased from 60 to 100%. The success rates of the 6 and 8 disconnection parts were 80% when *d* was 20 μm, while the rates were 0% when *d* were 40, 60, 80, and 100 μm. From photographs of broken ultrathin MEMS strain sensor films with 6 and 8 disconnection parts in Fig. 4, cleavages running between the disconnection parts where the high tensional strain to break the sensors film were found during the FEM analysis (the circled area of FEM results in Fig. 4). From comparing the numerical and experimental analyses, if 2*n* and *d* decreased to 4 and 20 μm, respectively, the success rate of chip-mounting will increase to 100% because the shear stress on those disconnection parts were concentrated for ease of separation, and the resultant small load on the sensor film reduced bending stress on the sensor film to avoiding breaking of it. Therefore, for 1 × 5 mm^2^ sensor film, 4 disconnection parts with *d* of 20 μm is an optimal design for highly successful plastic-scale-model assembly of an ultrathin MEMS strain sensor film.

**Figure 5:**
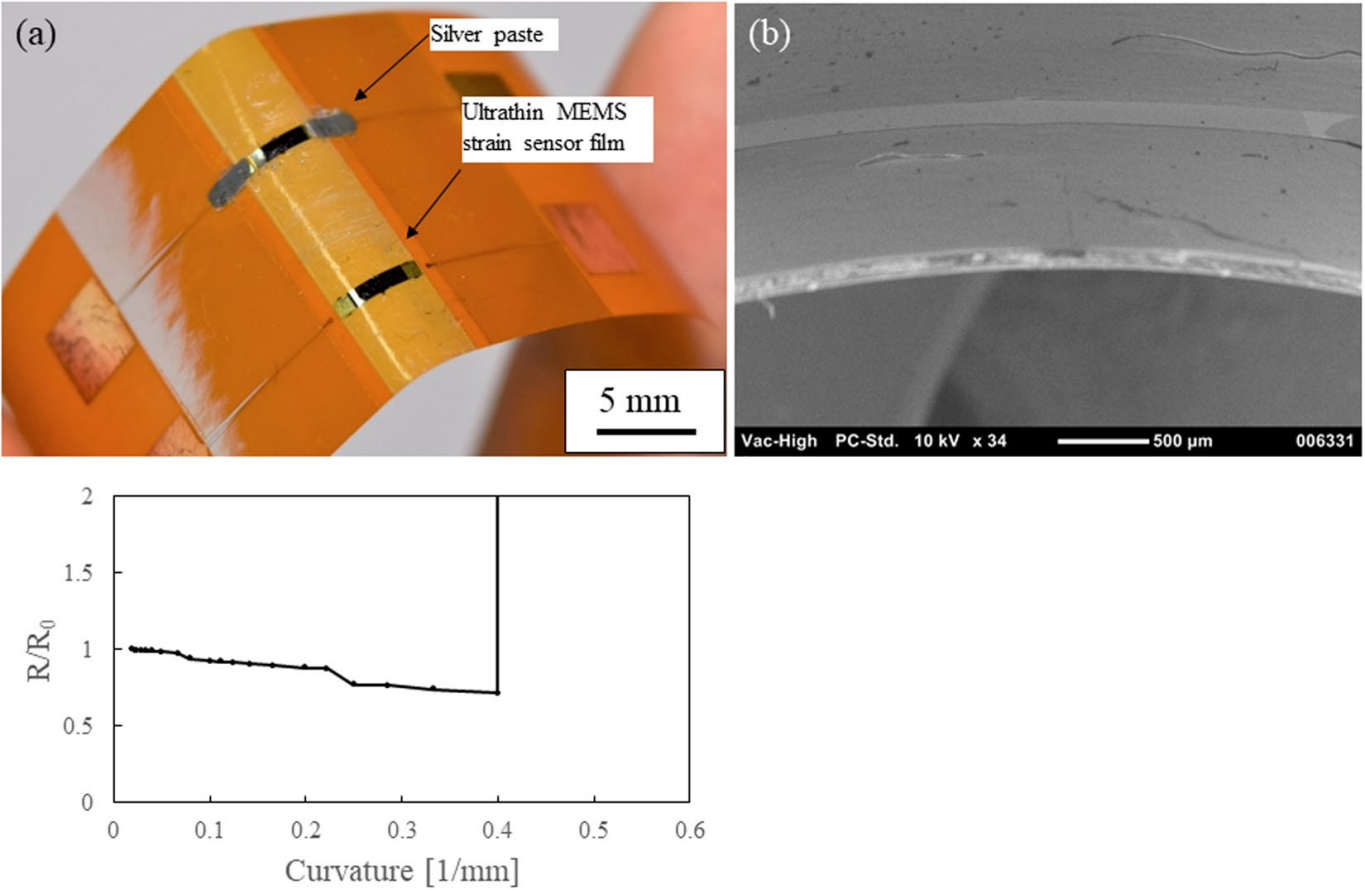
(**a**) Photograph of ultrathin MEMS strain sensor films, (**b**) SEM image of ultrathin MEMS strain sensor films, (**c**) Typical relationship between curvature and resultant electric resistance change of strain sensor film.

Figure 5(a,b) show a photograph and an SEM image of MEMS sensor films on a PI film under bending, respectively. The sensor film was only 5 μm thick; thus, it was highly flexible. Mechanical durability was investigated by conducting bending test. The both sides of sensor were connected to a multi-meter (Keithley 2400), and electrical resistances were measured when the 5 sensor films were bent to radii ranging from 50 to 2 mm. The bending curvature was tuned from 0.02 to 0.5 [1/mm] because the curvature is the multiplicative inverse of the bending radius. Figure 5(c) shows the typical relationship between bending curvatures and the resultant electric resistance change. The sensors sustained the curvature of 0.37 [1/mm], which is the averaged failure curvature of 5 sensor samples. The large curvature of 0.37 [1/mm] allow the sensors to be attached on human fingers to be applicable to wearable devices.

**Figure 6:**
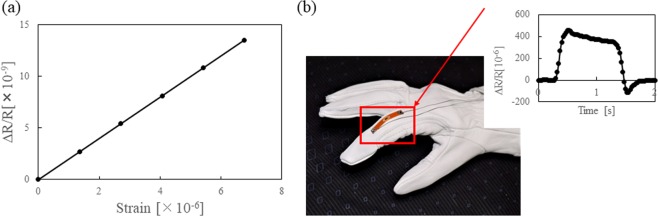
(**a**) Strain sensitivity of ultrathin MEMS strain sensors, (**b**) Human finger motion sensing with our ultrathin MEMS strain sensors.

We measured a gauge factor of our piezoresistive MEMS strain sensor film and demonstrated the human finger motion sensing with our sensor as a potential wearable application. The PI film with the strain sensor film was attached to a 0.5-mm-thick stainless-steel plate and small strain was applied to the stainless-steel plate for measuring the electric resistance change. The PI film with ultrathin MEMS strain sensor film was cut and placed on the stainless-steel plate by using glue (Loctite 496). Both sides of the stainless-steel plate were pulled with the force gauge and automatic moving stage (AIKOH engineering FTN-3001). The applied force ranged from 2 to 20 N, and the corresponding electric resistance of the MEMS strain sensor was measured with a Wheatstone bridge circuit, differential amplifier (NF circuit block corporation, model 5307), low pass noise filter (NF, model 3611), and stable electric power supply (NF, model 5394). The Wheatstone bridge circuit consists of two ultrathin MEMS strain sensor films and two dial electric resistance gauges (Sanhayato, DRB-6). The gain of the differential amplifier was 40, and cut off frequency and gain of the active low pass filter was 10 Hz and 10 times, respectively. The applied strain was calibrated using a commercial metal strain gauge (Kyowa Electronic Instruments Co., Ltd. 632-124). Figure 6(a) shows the relationship between applied strain and electric resistance change. The calculated gauge factor was at most 100, which is 33 times larger than with a conventional metal strain gauge and the same as with a semiconductor strain gauge. Therefore, a highly sensitive sensor can be fabricated and assembled with our plastic-scale-model assembly. Finally, we demonstrated the human finger motion sensing with our flexible ultrathin piezoresistive strain sensor. Figure 6(b) shows the photograph of the strain sensor attached on the human finger joint area of a glove. The graph in the Fig. 6(b) shows the electric resistance changed under human finger bending. Since the strain induced by bending is larger than that on the stainless-steel plate, larger electric resistance is observed. The advantages of our ultrathin MEMS strain sensor films are that it is thin (5 μm) and highly flexible while a conventional semiconductor strain gauge is thick (>100 um) and rigid.

## Discussion

We theoretically and experimentally analyzed our plastic-scale-model assembly with a conventional chip mounter for an ultrathin MEMS strain sensor film and found the optimal design of plastic-scale-model MEMS chips to achieve high yield of ultrathin MEMS sensor film mounting on a flexible substrate. To separate a MEMS sensor film and successfully mount it on a flexible substrate, the stress concentration of applied pressure on the disconnection parts for separation should be large, but the bending force on the sensor film should be small. From the mechanical characteristic model of ultrathin MEMS sensor film-mounting, *d* and 2*n* were found to be key parameters for successful sensor film separation. When 2*n* and *d* decreased from 8 to 4 and from 100 to 20 μm, respectively, the success rate increased. For 5-μm-thick 1 × 5 mm^2^ sensor film, the 4 disconnection parts with *d* of 20 μm achieved 100% success rate. Therefore, decreasing 2*n* and *d* is important for highly successful plastic-model MEMS-sensor assembly. The successfully fabricated MEMS sensor exhibited a high gauge factor of 100 and high flexibility. Therefore, our assembly with a conventional vacuum-suction chip mounter will lead to highly flexible and sensitive MEMS piezoresitive strain sensors toward wearable and flexible physical sensor applications.

## Methods

### Plastic-scale-model MEMS chip fabrication process

The plastic-scale-model MEMS chip fabrication is as follows. (1) The MEMS fabrication process starts with a SOI wafer made up of a 5-μm-thick device silicon layer, 1-μm-thick silicon dioxide layer, and 500-μm-thick handling silicon layer (SOI wafer, KST World Corporation). A phosphor ion is first implanted into the device silicon layer. After ion implantation, the surface of the device silicon layer is annealed at 950 °C with a lamp-annealing system to form a piezoresistive sensing layer. The phosphor-ion doping concentration is 10^20^. (2) Electrode films of Cr and Au are deposited and patterned on the device silicon layer. The device silicon layer is patterned and etched using the inductive coupled plasma reactive ion etching system (ICP-RIE) to define the sensor area and disconnection part. (3) After the backside silicon is patterned and etched using the ICP-RIE, the silicon dioxide layer is etched using the reactive ion etching system (RIE) to release the sensor area and disconnection parts of the device silicon layer.

### Ultrathin MEMS strain sensor film mounting process and wiring

By using a conventional vacuum-suction chip mounter (Hisol-model 400, Hisol Corporation), the chip mounter head separates the disconnection parts and places the sensor film on the desired area of the flexible circuit board. The chip mounter head vacuum-sucks the ultrathin MEMS strain sensor film. Specifically, the position of the ultrathin MEMS strain sensor film is first detected with the microscope camera of the chip mounter and the chip mounter head is moved above the sensor film. The chip mounter head first moves down without vacuum and breaks the disconnection parts of the MEMS chip. Then vacuum suction starts, and the sensor film is picked up by the chip mounter head. After the chip mounter head moves to the desired position on the flexible substrate, the sensor film is taken down slowly. The chip mounter head releases the sensor film by stopping the suction. Then, the adhesive glue, which is stronger than PDMS adhesive, is used to release the sensor films and fixes the sensor film on the substrate. Silver paste (PE873, Dupont) is then coated between the Au pads of the sensor film and Cu electrode on the flexible circuit board.
